# Targeting phosphoinositide 3-kinase (PI3K) in head and neck squamous cell carcinoma (HNSCC)

**DOI:** 10.1186/s41199-018-0030-z

**Published:** 2018-06-04

**Authors:** Kyungsuk Jung, Hyunseok Kang, Ranee Mehra

**Affiliations:** 1 Department of Medicine, Fox Chase Cancer Center, 333 Cottman Ave, Philadelphia, PA USA; 2 Department of Oncology, The Sidney Kimmel Comprehensive Cancer Center at Johns Hopkins, 201 N Broadway, Baltimore, MD USA

**Keywords:** HNSCC, PI3K, mTOR, Akt, EGFR, *PIK3CA*, HPV, Drug resistance, Precision medicine

## Abstract

The landscape of head and neck squamous cell carcinoma (HNSCC) has been changing rapidly due to growing proportion of HPV-related disease and development of new therapeutic agents. At the same time, there has been a constant need for individually tailored treatment based on genetic biomarkers in order to optimize patient survival and alleviate treatment-related toxicities. In this regard, aberrations of PI3K pathway have important clinical implications in the treatment of HNSCC. They frequently constitute ‘gain of function’ mutations which trigger oncogenesis, and PI3K mutations can also lead to emergence of drug resistance after treatment with EGFR inhibitors. In this article, we review PI3K pathway as a target of treatment for HNSCC and summarize PI3K/mTOR inhibitors that are currently under clinical trials. In light of recent advancement of immune checkpoint inhibitors, consideration of PI3K inhibitors as potential immune modulators is also suggested.

## Background

Head and neck squamous cell carcinoma (HNSCC) arises from mucosal epithelium of oral cavity, pharynx and larynx. An estimate of 61,000 new cases of HNSCC were diagnosed in the US in 2016, with 13,190 deaths attributable to the disease [1]. Traditional risk factors include tobacco smoking, alcohol consumption, betel nut chewing and genetic predisposition such as Fanconi anemia [2–4]. Human papillomavirus (HPV) has recently emerged as a major and distinct risk factor for HNSCC. HPV-related HNSCC most commonly arises in oropharynx and has been associated with younger age of disease onset, less smoking history, better performance status and favorable prognosis [5]. The proportion of HPV-positive oropharyngeal squamous cell cancer has significantly increased for the past decade regardless of sex and race [6], raising the need for a separate therapeutic strategy.

Comprehensive genomic analysis of HNSCC revealed frequent alterations in genes encoding molecules in phosphoinositide 3-kinase (PI3K) pathway including *PIK3CA*, *PTEN* and *PIK3R1* [7, 8]. In particular, HPV-related HNSCC frequently harbors mutations in the helical domain of *PIK3CA*, yet its biological significance has not been fully elucidated. In the era of precision medicine, it is becoming more important to understand key genomic alterations and their therapeutic implications [9]. This review will focus on the role of PI3K-Akt-mTOR pathway in relation to epidermal growth factor receptor (EGFR) and their clinical applications in HNSCC.

## Phosphoinositide 3-kinase (PI3K) and PI3K-Akt-mTOR pathway

**Fig. 1:**
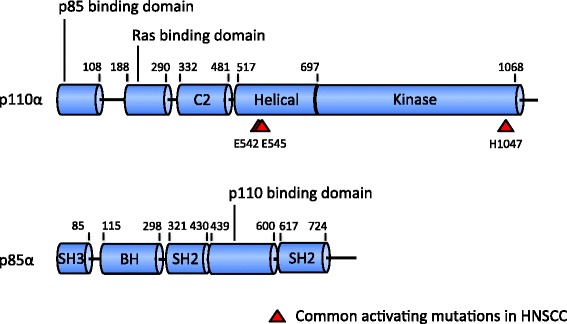
Linear composition of p110α and p85α molecules. Red arrowheads in p110α indicate ‘hotspot’ mutations. C2 in p110α is a putative membrane-binding domain. Breakpoint cluster region-homology (BH) domain in p85α has shown GTPase activating protein (GAP) activity toward Rab family. Rab GTPase induces degradation and deregulation of activated growth factor receptors, and mutated Rab GAP induces cell transformation [148]. However, it is unclear if this function is still active in complex with p110α [149]. BH domain in p85α is flanked by proline-rich domain, implying an auto-regulatory mechanism in interaction with its SH3 domain [150]

PI3K is a family of phospholipid kinase that is divided into three classes based on structure, function and substrate specificity. Class I PI3K is a heterodimer that consists of a regulatory and a catalytic subunit. It is further divided into class IA and IB. For class IA PI3K, there are three variants of catalytic subunit, p110α, p110β and p110δ (encoded by *PIK3CA*, *PIK3CB* and *PIK3CD*), and five variants of regulatory subunit, p85α, p55α, p50α (encoded by *PIK3R1* and splice variants), p85β and p55δ (encoded by *PIK3R2* and *PIK3R3*). p85 regulatory subunit contains Src homology 2 (SH2) domain which binds to phosphorylated Y-X-X-M motif in receptor tyrosine kinase [10]. It was found that five isoforms of regulatory subunit express different affinities to tyrosine kinases [11], and each p110 subunit is selectively recruited to receptor activation [12, 13]. These findings are consistent with selective mutation of p110 in various types of cancer and provides important prospect for targeted therapy. *PIK3CA* is one of the most commonly mutated and extensively studied oncogenes in various types of human cancer. An analysis of The Cancer Genome Atlas (TCGA) data showed that *PIK3CA* was the most frequently mutated gene in breast cancer samples, second most frequently mutated gene in uterine corpus endometrial cancer and third most commonly mutated gene in HNSCC [14]. *PIK3CA* is also heavily mutated in lung squamous cell carcinoma, urothelial carcinoma of bladder and colorectal adenocarcinoma [14]. Molecular composition of p110α, the product of *PIK3CA*, and p85α are illustrated in Fig. 1.

Class IB PI3K consists of p110γ catalytic subunit (encoded by *PIK3CG*) and p101 or p87 regulatory subunit (encoded by *PIK3R5*, *PIK3R6*). Class IA and IB PI3K phosphorylate 3-hydroxyl group of phosphatidylinositol (PI), phosphatidylinositol 4-phosphate (PIP) and phosphatidylinositol 4,5-bisphosphate (PIP2), producing phosphatidylinositol 3-phosphate (PI-3-P), phosphatidylinositol 3,4-bisphosphate (PI-3,4-P2) and phosphatidylinositol 3,4,5-triphosphate (PIP3), respectively [15]. Expressions of p110δ and p110γ are found exclusively in lymphocytic immune system whereas p110α and p110β are expressed ubiquitously [16]. Idelalisib, a drug used for treatment of lymphoma, is a selective inhibitor of p110δ which is abundantly expressed in malignant B cells [17].

Class II PI3K is a monomer of catalytic isoforms, C2α, C2β and C2γ (encoded by *PIK3C2A*, *PIK3C2B* and *PIK3C2G*), and lacks regulatory subunit. Class II lipid kinase produces PI-3,4-P2 from PIP and PI-3-P from PI. C2α isoform found in endosomes was suggested to play a role in angiogenesis and vascular barrier formation [18]. Class III PI3K is a heterodimer of a regulatory (Vps15, encoded by *PIK3R4*) subunit and a catalytic subunit (Vps34, encoded by *PIK3C3*), which converts PI to PI-3-P. Little is known about physiologic role of class III PI3K, but it was implicated in induction of autophagy in the state of nutrient deficiency [19].

The family of PI3K proteins mainly regulates cellular growth and cycle. Its activation is triggered by upstream receptor tyrosine kinase such as ErbB family receptor (including EGFR), platelet-derived growth factor receptor (PDGFR), insulin-like growth factor 1 receptor (IGF-1R) or G protein-coupled receptor (GPCR). PI3K attaches a phosphate group to the 3′ hydroxyl of the inositol head of PIP2, converting it to PIP3 [20]. Inositol phospholipids constitute a minor part of the cellular membrane and phosphorylation of inositol head has little effect on membrane structure. However, phosphorylated inositol head protruding from the membrane provides an anchoring site for secondary signaling molecules that are floating in the cytosol. Once PIP3 is formed by PI3K, cytosolic molecules such as Akt/Protein kinase B localize to plasma membrane and become tethered to the head of PIP3 via Pleckstrin homology (PH) domain in N terminal [21]. Activated Akt, in turn, phosphorylates a series of molecules including mechanistic target of rapamycin (mTOR) that promotes cell survival, proliferation and motility. The action of PI3K, conversion of PIP2 to PIP3, is negatively regulated by reverse phosphatases, such as phosphatase and tensin homolog (PTEN). Other cytoplasmic molecules that contain PH domain and interact with PIP3 include Rho-guanine nucleotide exchange factor (GEF). Rho family proteins, when activated by GEF, remodel cytoskeleton, decrease contact inhibition and increase cell motility, all of which elevate invasiveness in cancer cells [22].

## Implications of PI3K pathway alteration for EGFR pathway in HNSCC

**Fig. 2:**
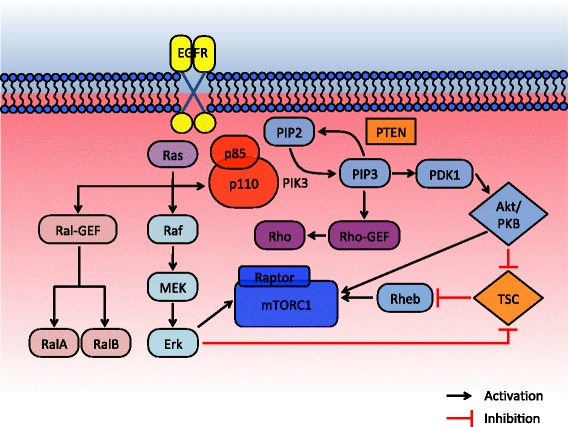
Interactive signaling pathway of EGFR-PI3K-mTOR. PI3K binds to cytoplasmic tail of receptor tyrosine kinase via SH domains within p85 regulatory subunit. Activation signal can also be transferred through Ras-binding domain in p110 catalytic subunit which tethers PI3K molecule to Ras protein in growth receptors. p110 activation by Ras binding is inhibited by p85 subunit which can be released by co-stimulation of SH domain by tyrosine kinase [151]

EGFR is a cell surface receptor tyrosine kinase in ErbB family and has been an attractive therapeutic target for various human cancers including HNSCC. The receptor becomes activated by ligand binding which transitions EGFR monomers to the allosteric homodimer. Receptor dimerization stimulates tyrosine kinase activity in C terminal domain and initiates downstream phosphorylation cascade through PI3K-Akt-mTOR, Raf-MEK-MAP kinase or JAK/STAT pathways (Fig. 2).

It has been well known that EGFR overexpression is involved in carcinogenesis of HNSCC [23, 24], and associated with poor prognosis [25, 26]. EGFR-targeting strategy with a monoclonal antibody, cetuximab, has prolonged survival of patients with locally advanced HNSCC in combination with radiotherapy [27]. Cetuximab is currently used with platinum-based chemotherapy as the first line treatment for HNSCC or for recurrent or metastatic (R/M) disease [28, 29]. However, efforts to develop a predictive biomarker for EGFR-targeting treatment have not been successful. In particular, overexpression of EGFR assessed by immunohistochemistry (IHC) could not be correlated with the level of treatment response to cetuximab [30–32]. Additionally, resistance to cetuximab has been widely observed in various types of cancer including HNSCC. Several evasive mechanisms may serve to restore original oncogene dependence, circumventing the initial targeting treatment. Receptors can potentially abrogate inhibitory action of therapeutic agents as they obtain second mutations that result in pharmacokinetic changes [33]. A well-known mutation of EGFR, T790M, enhances affinity of the kinase pocket for ATP, which competitively blocks binding of tyrosine kinase inhibitors [34]. Copy number gains of target genes also reactivate dependent pathway and counteract the treatment effect. For example, amplification of *BRAF* via copy number gains was found in 8% of the tumor samples from metastatic melanoma treated with BRAF inhibitors [35]. Studies with HNSCC demonstrated as well that copy number alteration by amplification of 7p11.2 accounts for a number of cases of EGFR activation [36–38]. It was also hypothesized that ligand overexpression or receptor cross phosphorylation triggers uncontrolled EGFR hyperactivity. A genetic profiling of HNSCC samples with EGFR activation revealed that EGFR ligands (including TGFα) were highly expressed in a subset, suggesting an establishment of an autocrine loop [39].

Alternatively, the function of target gene can be bypassed by activating downstream molecules of the signaling cascade or switching dependence to an alternative pathway for cell growth and proliferation [40]. As the tumor progresses and develops genomic heterogeneity, cells with genetic survival benefit outgrow through evolutionary selection pressure. In consistent with this theory, whole-exome sequencing of melanoma cells that are resistant to BRAF inhibitor revealed diverse genetic alterations in the downstream MAPK pathway [41]. Similarly, *KRAS* amplification or mutation was found in tumor samples from colorectal cancer patients who developed resistance to EGFR inhibitors [42]. Relevant to our review, compensatory activation of downstream pathway, mainly PI3K, has been proposed as one of the major resistance mechanisms to EGFR inhibitors in HNSCC. Gene expression of the molecules in PI3K pathway was elevated in cetuximab-resistant strains compared to cetuximab-susceptible cells [43], and addition of mTOR/PI3K inhibitor effectively achieved control of cell growth in HNSCC that acquired resistance to EGFR inhibitors [44, 45].

## PI3K-mTOR alteration in HNSCC

66% of HNSCC harbor genomic alterations in one of the major components of PI3K pathway [46]. An analysis of whole-exome sequencing of 151 HNSCC tumors revealed that PI3K is the most commonly mutated mitogenic pathway among PI3K, JAK/STAT and MAPK and that presence of multiple mutations in PI3K signaling pathway is correlated with more advanced disease [8]. Physiologic data confirms that an aberrant PI3K-mTOR pathway is associated with cell motility, invasion and metastasis. PI3K-PTEN balance has a direct effect on chemotaxis and cell motility as it controls actin cytoskeleton via Rho family proteins, such as Rho, Rac and CDC42 [22, 47]. PIP3 and PIP2 determine epithelial polarity in individual cells, thus dysfunctional PI3K results in epithelial-mesenchymal transition, a critical event in tumor invasion [48].

*PI3KCA* is among the most frequently mutated genes in HNSCC, affected both in HPV-positive and negative diseases (56 and 34%, respectively) [7]. *PIK3CA* mutations in HPV-positive HNSCCs are concentrated in helical domain, whereas mutations are more spread out in HPV-negative diseases [9, 49]. TCGA data presents that 73% of *PIK3CA* mutations are located at E542, E545 in the helical domain and in H1047 in the kinase domain [7]. Frequency of these ‘hotspot’ mutations is also higher in HPV-positive oropharyngeal cancers [50].

Targeting *PIK3CA* alteration in human squamous cell xenografts has demonstrated susceptibility to treatment in vitro and in vivo, leading a path for its clinical implication. Inhibition of PI3K by competitive blockage of ATP binding site led to decreased phosphorylation of Akt in several studies [51–54]. In a number of the patient-derived xenografts harboring E545K and H1047R mutations, PI3K inhibitors were effective in achieving control of tumor growth [43, 55, 56]. Additionally, activation of PI3K/mTOR pathway from either mutation or gene amplification was positively correlated with tumor susceptibility to PI3K inhibitors in xenograft models [52, 57–59]. However, preclinical data also suggested that additional molecular change should interact with *PIK3CA* alteration for tumorigenesis. Cell lines engineered to harbor *PIK3CA* mutations in the ‘hotspots’ responded more favorably to PI3K/mTOR dual inhibition than PI3K inhibition only, indicating that tumor survival is not strictly dependent on the activated PI3K [60]. In a similar sense, PI3K inhibition demonstrated markedly synergistic effect when combined with EGFR or MEK inhibition [61]. Interestingly, *PIK3CA* activation in HPV-positive HNSCC did not necessarily lead to increased Akt target phosphorylation, but instead, led to increased mTOR activity and showed more sensitivity to PI3K/mTOR dual inhibition than Akt inhibition [62]. This finding can be extended to more favorable efficacy of PI3K/mTOR inhibitors over Akt inhibitors in clinical settings [63].

Locations of mutations affect PI3K structure and function, resulting in different responsiveness to inhibition and clinical outcome. Regulatory subunit p85 normally suppresses catalytic function of p110 at resting stage. Consequently, C terminal truncation or internal deletion of p85 releases p110 from negative regulation and constitutively activates the PI3K pathway [64, 65]. Additionally, as frequently mutated E542 and E545 in p110 are located at a distance from the kinase domain, it is plausible that mutations at these spots alter regulatory control of p85. Indeed, E545K mutation in the helical domain of p110 changes acid-base charge and disrupts inhibitory interaction between p85 and p110 [66]. H1047R mutation in the kinase domain, on the other hand, shifts orientation of the residue and changes conformation of the two loops of kinase that contact cell membrane. This allows for kinase access to phospholipid that is less regulated by p85 [67].

Independently from p110, p85 as a monomer also down-regulates PI3K activation: p85 is naturally more abundant than p110 and excess p85 monomers can sequestrate insulin receptor substrate 1 (IRS-1), an adaptor molecule that mediates signal transduction between IGF-1R and downstream PI3K [68]. Thus, in wild-type cells, the p85 monomer competes with the p85-p110 dimer for IRS binding and signal transduction. In heterozygous knock out cells, the amount of p85 monomers decreases more than p85-p110 dimers which up-regulates the PI3K pathway [69]. However, in null cells, complete absence of regulatory subunit to stabilize p110 leads to significantly decreased signal transduction causing cell apoptosis [69]. Although not as frequent as in *PIK3CA*, mutations in *PIK3R1* (encoding p85α) can be found in 3% of HPV-positive HNSCC and 1% of HPV-negative HNSCC according to TCGA data [7].

Alteration of *PTEN* tumor suppressor gene is among the frequently found somatic mutations in human cancers as well as germline mutations causing hereditary cancer syndromes. PTEN dephosphorylates PIP3 to PIP2, inhibiting mitogenic signal transduction in the PI3K pathway. PTEN also interacts with PI3K, which plays a key role in chemotaxis and tumor metastasis [47, 48]. Clinical data has shown that loss of *PTEN* expression is a poor prognostic marker in oral squamous cell cancer [70]. However, *PTEN* loss was found in only a small number of HNSCC (8.16%), implying that it is a relatively minor component in PI3K pathway activation [8].

## Targeting PI3K-Akt-mTOR pathway in clinic

### PI3K inhibitor

#### Buparlisib (BKM120)

Buparlisib is an orally bioavailable pan-PI3K inhibitor, targeting the ATP binding site of p110 kinase domain. Its inhibitory potency is equitable on class IA isoforms of p110α, β and δ, but slightly less against class IB p110γ [51]. An in vitro study demonstrated IC_50_ values for Akt inhibition of 104 ± 18, 234 ± 47 and 463 ± 87 nmol/L for PI3Kα, β and δ, respectively [51]. Buparlisib is rapidly absorbed orally and its serum concentration increases proportionately to dosage [71]. The molecule also penetrates blood brain barrier and administration of buparlisib by gavage effectively controlled metastatic growth of human breast cancer in mouse brain [72]. Based on preclinical data, its antitumor activity was also attributed to suppression of microtubular dynamics [73], and antiangiongenic effect [51]. A combination of buparlisib, cetuximab and radiation exerted a synergistic antiproliferative effect on human head and neck cancer cell lines [74, 75]. In vivo, buparlisib inhibited PI3K activity in cell lines with wild-type *PIK3CA* as well as mutant form harboring any hotspot mutation of E542K, E545K or H1047R [76]. In a phase I dose-escalation study for advanced solid tumors, most common side effects included rash, abnormal hepatic function, alteration in glucose metabolism and fatigue [71]. In a recent randomized phase II trial with R/M HNSCC, adding buparlisib to paclitaxel improved progression-free survival (PFS) to 4–6 months compared to 3–5 months in the placebo plus paclitaxel group (*p* = 0.011) [77]. In this trial, comparable proportions of the patients had a mutation in *PIK3CA*, 11% and 13% in the buparlisib and control arm, respectively. Patients taking buparlisib also maintained stable quality of life and demonstrated good tolerance to the treatment compared to the placebo group, as similar proportions of patients discontinued the treatment due to adverse effects [77]. However, this study failed to demonstrate significant improvement in overall survival (OS) with buparlisib partly because of insufficient power. There are several ongoing clinical trials to evaluate the efficacy and safety of buparlisib with or without additional therapy (Table 1).

**Table 1:** Clinical trials evaluating PI3K or mTOR inhibitor in patients with HNSCC

Agent	Clinical Trial Identifier	Other Targeted Agent	Additional Therapy	Conditions	Phase	Status
PI3K inhibitor						
Alpelisib (BYL719)	NCT02145312	–	–	R/M HNSCC, failed to respond to platinum-based therapy	II	Not yet recruiting
	NCT02537223	–	Cisplatin, radiation	Locoregionally advanced HNSCC, not previously treated	I	Active, recruiting
	NCT01602315	Cetuximab	–	R/M HNSCC	I/II	Terminated (sponsor withdrawal)
	NCT02298595	Cetuximab	Cisplatin	HPV-associated oropharyngeal SCC	I/II	Not yet recruiting
Buparlisib (BKM120)	NCT01816984	Cetuximab	–	R/M HNC	I/II	Active, not recruiting
	NCT01737450	–	–	Recurrent or progressive HNC	II	Active, recruiting
	NCT02113878	–	Cisplatin, radiation	Locally advanced HNSCC	I	Active, recruiting
PX-866	NCT01252628	Cetuximab		R/M HNSCC	II	Completed
	NCT01204099	Docetaxel		Locally advanced or R/M HNSCC	II	Completed
Copanlisib	NCT02822482	Cetuximab	–	HNSCC with PI3KCA mutation/amplification or PTEN loss	I/II	Active, recruiting
INCB050465	NCT02646748	Itacitinib	Pembrolizumab	Advanced solid tumors	I	Active, recruiting
mTOR inhibitor						
Sirolimus	NCT01195922	–	–	Advanced HNSCC, not previously treated	I/II	Completed
Temsirolimus	NCT01172769	–	–	R/M HNSCC	II	Completed
	NCT01009203	Erlotinib	–	Advanced HNSCC, refractory to platinum	II	Terminated (high patient withdrawal rate)
	NCT01016769	–	Paclitaxel, carboplatin	R/M HNSCC	I/II	Active, not recruiting
	NCT02215720	Cetuximab	–	Advanced or metastatic solid tumors	I	Active, recruiting
	NCT00703625	–	Docetaxel	Resistant solid malignancies	I	Completed
Everolimus (RAD001)	NCT01332279	Erlotinib	Radiation	Recurrent HNC, previously treated with radiation	I	Withdrawn (sponsor withdrawal)
	NCT01313390	–	Docetaxel	R/M HNSCC	I/II	Terminated (lack of recruitment)
	NCT01009346	Cetuximab	Cisplatin, carboplatin	R/M HNSCC	I/II	Terminated (toxicity)
	NCT01051791	–	–	R/M HNSCC	II	Active, not recruiting
PI3K/mTOR dual inhibitor						
SF1126	NCT02644122	–	–	R/M HNSCC	II	Active, recruiting
Gedatolisib	NCT03065062	Palbociclib	–	Advanced HNSCC	I	Active, recruiting
Dactolisib (BEZ235)	NCT00620594	–	–	Advanced solid tumors	I	Completed
PI3K/HDAC dual inhibitor						
CUDC-907	NCT02307240	–	–	Advanced or relapsed solid tumors	I	Active, recruiting

#### PX-866

PX-866 is an analog of wortmannin that irreversibly inhibits class I PI3K by binding to Lys in ATP catalytic site [78]. Potent and irreversible binding of PX-866 enables sub-nanomolar IC_50_ values of 0.1, 1.0 and 2.9 nmol/L for PI3Kα PI3Kγ and PI3Kδ, respectively, in contrast to much higher IC_50_ of > 300 nmol/L for PI3Kβ [79]. In vivo studies revealed antitumor activities of PX-866 against human colon cancer, ovarian cancer and lung cancer xenografts [80]. It enhanced antitumor activities of cisplatin and radiation treatment in colon cancer and ovarian cancer cells, respectively [80]. PX-866 also effectively overcame resistance to EGFR inhibitor in human lung cancer cells lacking expression of ErbB-3 [79]. PX-866 induced cessation of tumor growth in xenograft models of human HNSCC which included one case of *PIK3CA* gene amplification and another case of E545K [43]. However, clinical trials of PX-866 failed to show promising results. In phase II clinical trials, combined use of PX-866 with either cetuximab or docetaxel failed to achieve improved PFS or OS compared to each treatment alone [81, 82].

#### Alpelisib (BYL719)

Theoretically, a selective inhibitor of PI3Kα can achieve antitumor activity without affecting other isoforms of PI3K, allowing for a more favorable side effect profile. Alpelisib was designed as a specific inhibitor of PI3Kα, the product of frequently mutated *PIK3CA* [83]. The molecule inhibits wild-type PI3Kα (IC_50_ = 4.6 nmol/L) as well as PI3Kα with common *PI3KCA* mutations, such as E545K or H1047R (IC_50_ = 4 nmol/L), more potently than PI3Kδ (IC_50_ = 290 nmol/L) or PI3Kγ (IC_50_ = 250 nmol/L) [52]. Preclinical data also suggested that *PIK3CA* mutation makes cancer cells more vulnerable to PI3K inhibition by alpelisib. In vitro pharmacologic sensitivity screen among a broad panel of cancer cell lines revealed that sensitivity to alpelisib was positively associated with the presence of *PIK3CA* mutation, amplification or copy number gain [84], which was confirmed by an in vivo study using mouse models [52]. In a HNSCC cell line (Cal-33) and a patient-derived xenograft model, both harboring H1047R mutation in *PIK3CA*, administration of alpelisib using nanoparticles induced inhibition of tumor growth and sensitization to radiation [55]. Compared to HNSCC cell lines with wild-type *PIK3CA*, cell lines with *PIK3CA* H1047R mutation were more susceptible to antiproliferative effect of alpelisib [56]. In another in vivo study, *PIK3CA* mutation, regardless of its location, was the strongest predictive feature that correlated with favorable response to alpelisib [52]. Compensatory hyperactivation of *PIK3CA* is one of the major mechanisms of treatment resistance, thus PI3K inhibitors are being tested with other targeted therapies, such as EGFR inhibitors. Inhibition of PI3K with alpelisib enhanced tumor sensitivity to cetuximab in HNSCC xenograft models [85]. A phase I trial of alpelisib combined with cetuximab in R/M HNSCC resulted in one partial response (PR), three unconfirmed PRs and five stable diseases (SDs) among 32 cases with relatively good patient tolerance [86]. PI3K activation status was unknown in this trial. In a more recent phase I trial of alpelisib, any of complete response (CR), PR or SD was achieved in 13 out of 19 study participants with *PIK3CA*-mutant HNSCC (NCT01219699) [87].

#### Copanlisib

Copanlisib is a potent inhibitor of class I PI3K with sub-nanomolar IC_50_. The molecule exhibits preferential activity against PI3Kα and PI3Kδ over PI3Kβ and PI3Kγ (IC_50_ values of 0.5 and 0.7 nmol/L over 3.7 and 6.4 nmol/L, respectively) [57, 88]. It demonstrated superior inhibitory effect in cells with *PIK3CA* activating mutations over wild-type in breast cancer and non-small cell lung cancer xenografts [57]. Phase I trials in patients with advanced or refractory solid tumors presented good patient tolerance and evidence of disease control [89, 90]. Efficacy and safety of combined copanlisib and cetuximab for HNSCC is under study (NCT02822482).

### mTOR inhibitor

#### Sirolimus (rapamycin)

Sirolimus was initially developed as an antifungal metabolite, extracted from the bacterium *Streptomyces hygroscopicus* [91]. However, since its immunosuppressive and antiproliferative properties were revealed, this macrolide molecule has been more widely used for oncologic treatment and for prevention of graft rejection or coronary stent blockage. Sirolimus binds with FKBP12 (12 kDa FK506-binding protein) to form a gain-of-function complex that function as an inhibitor of mTOR complex 1 (mTORC1) [92]. This compound, as a result, inhibits metabolic alteration and cell proliferation which is triggered by upstream gain-of-function mutations, such as PI3K and Akt. Sirolimus demonstrated antiproliferative activity in HNSCC cell lines inducing synergistic effect with chemotherapeutic agents or radiation [93, 94]. In HNSCC xenograft models with activated PI3K-Akt pathway, administration of sirolimus induced marked inhibition of tumor growth and cell apoptosis [58, 59]. It also suppressed lymphangiogenesis in HNSCC xenograft models and prevented spread of the cancer cells to adjacent lymph nodes [95]. In a phase I trial of sirolimus and bevacizumab for patients with advanced malignancies, no objective response was observed among the participants with HNSCC [96]. However, among the patients with stage II-IVA, untreated HNSCC, neoadjuvant trial of sirolimus followed by definitive therapy (surgery or chemoradiation) demonstrated significant clinical responses (one CR, one PR and 14 SDs among 16 patients) with good patient tolerance [97]. Sirolimus is known for poor bioavailability and low predictability of serum concentration after intestinal absorption, thus its narrow therapeutic window and a long half-life require regular drug concentration monitoring [98]. Based on these concerns, analogs of sirolimus have been developed to improve pharmacokinetic properties.

#### Temsirolimus

Temsirolimus is a water-soluble analog of sirolimus and can be administered parenterally [99]. It undergoes hydrolysis after administration to form sirolimus, but the medication itself is also capable of inhibiting mTOR. Temsirolimus is currently FDA approved for the treatment of advanced renal cell carcinoma [100]. Several preclinical studies proved that a combination of temsirolimus and cetuximab induces synergistic antitumor effect, as it mitigates or prevents compensatory downstream mTOR over-activation induced by EGFR inhibitor [101–105]. There have been a number of phase I/II trials using temsirolimus in patients with HNSCC. In a phase I study of temsirolimus used with carboplatin and paclitaxel in R/M HNSCC, 22% of the patients exhibited objective PRs [106]. The information regarding PI3K activation status was lacking in this study. In TEMHEAD trial, a phase II study of temsirolimus in R/M HNSCC refractory to platinum and cetuximab, tumor shrinkage occurred in 39.4% of the patients mostly within the first six weeks of the treatment. However, no objective response was achieved, nor did *PI3KCA* mutational status (H1048Y and G1050S) predict treatment success [107]. In another trial including a broad range of advanced malignancies, the combination of bevacizumab, cetuximab and temsirolimus was effective in achieving PRs in 25% of the patients with HNSCC, but a few patients were withdrawn from the trial because of toxicities [108]. In this study, treatment-responders did not carry *PIK3CA* mutation in HNSCC cells. A trial combining temsirolimus with erlotinib for R/M HNSCC was closed early due to toxicity and patient death [109]. In a phase I pharmacokinetic study of temsirolimus, dose-limiting toxicities occurred such as thrombocytopenia, stomatitis or mucositis, asthenia, manic-depressive syndrome and rash [110]. Thus, the treatment effect of temsirolimus should be evaluated against potential toxicities and more clinical trials are ongoing.

#### Everolimus (RAD001)

Everolimus is a hydroxyethyl derivative of rapamycin, offering improved oral bioavailability. The medication has a short half-life, allowing for quick establishment of stable status and improved drug safety [111]. After intestinal absorption, everolimus is not converted to rapamycin, instead forms a complex with FKBP12 and inhibits mTOR [112]. It is currently approved by the FDA for treatment of multiple malignancies including advanced breast cancer, kidney cancer, neuroendocrine tumor (NET) of pancreas, progressive NET of GI and lung, tuberous sclerosis-associated renal angiomyolipoma and subependymal giant cell astrocytoma [113]. Although everolimus was effective in arresting tumor growth in HNSCC xenograft models [114, 115], clinical data was not as encouraging. Several phase I studies demonstrated PRs among patients with HNSCC [116–119], but the doses of everolimus used were different depending on other treatments combined, such as platinum, docetaxel, cetuximab or radiation. Phase II trials with everolimus also failed to demonstrate clinical benefit for HNSCC. Either as monotherapy or combination with erlotinib, treatment with everolimus was not successful in achieving objective response in patients with previously treated R/M HNSCC [120, 121]. There is a currently active clinical trial testing everolimus monotherapy in patients with R/M HNSCC (NCT01051791).

### PI3K/mTOR dual inhibitor

#### SF1126

SF1126 is a peptide-conjugated prodrug of LY294002, with improved water solubility and pharmacokinetics. RGDS conjugation enables the molecule to bind to specific integrins within the tumor, enhancing drug permeability [53]. LY294002 is a pan-PI3K inhibitor, with IC_50_ values of 720 nmol/L, 306 nmol/L, 1.33 μmol/L and 1.6 μmol/L for PI3Kα, PI3Kβ, PI3Kδ and PI3Kγ respectively, and similar IC_50_ for mTOR (1.5 μmol/L) [53, 122]. In a phase I trial, SF1126 as a single agent was effective in maintaining stable diseases in patients with GIST and clear cell renal cancer, and in combination with rituximab decreased absolute lymphocyte count and lymph node/spleen size in CLL [123]. SF1126 monotherapy is now being evaluated for treatment of R/M HNSCC (NCT02644122).

#### Gedatolisib

Gedatolisib is a potent and reversible inhibitor of class I PI3K and mTOR. IC_50_ values for PI3Kα, PI3Kβ, PI3Kδ, PI3Kγ and mTOR are 0.4 nmol/L, 6 nmol/L, 8 nmol/L, 6 nmol/L and 10 nmol/L, respectively [124]. The inhibitory activity against PI3Kα with hotspot mutations, such as E545K and H1047R, are comparatively low (0.6 nmol/L and 0.8 nmol/L) [124]. Its antitumor activity was demonstrated in in vitro studies using mutant cells harboring E545K or H1047R in *PIK3CA* as well as wild-type [124, 125]. Gedatolisib also inhibited cell proliferation and increased radiosensitivity of human nasopharyngeal cancer cells with PI3K/mTOR hyperactivation [126]. Additionally, use of gedatolisib in EGFR inhibitor-resistant HNSCC suppressed cell survival and induced apoptosis [45]. Phase I trials with gedatolisib for patients with advanced cancer demonstrated potential antitumor activities with PRs and acceptable tolerance [127, 128]. However, no apparent relationship between *PIK3CA* alteration and treatment response was observed in these trials. There is an ongoing phase I trial of gedatolisib combined with palbociclib (CDK4/CDK6 inhibitor) for advanced solid tumors including HNSCC (NCT03065062).

#### Dactolisib (BEZ235)

Dactolisib is an ATP-competitive dual inhibitor of PI3K and mTOR, exerts more potency on PI3Kα, PI3Kδ, PI3Kγ and mTOR (IC_50_ values of 4, 7, 5 and 21 nmol/L, respectively) than PI3Kβ (IC_50_ = 75 nmol/L) [54, 129]. Dactolisib exhibited potent antiproliferative activity, halting cell cycles at G1 [54] and attenuating VEGF expression [129]. HNSCC cell lines with H1047R mutation were more susceptible to inhibition with lower IC_50,_ whereas E545K conferred only slightly increased sensitivity [60]. In clinical settings, however, there has been little evidence to support drug efficacy and safety. When dactolisib was used for patients with castration-resistant prostate cancer or everolimus-resistant pancreatic NET, the trials were discontinued due to dose-limiting toxicities, such as stomatitis, vomiting, diarrhea or hyperglycemia [130, 131]. Combination of dactolisib and everolimus tested in patients with various advanced solid tumors, including one case of HNSCC, failed to demonstrate objective response [132]. Another phase I trial of dactolisib treatment for various, advanced solid tumors is now complete and the result is being awaited (NCT00620594).

### PI3K/HDAC dual inhibitor

#### CUDC-907

CUDC-907 is an orally administered inhibitor of class I PI3K isoforms and histone deacetylase (HDAC). IC_50_ values for PI3Kα, PI3Kβ, PI3Kδ and PI3Kγ are 19, 54, 38 and 311 nmol/L, respectively [133]. Simultaneous inhibition of PI3K and HDAC has demonstrated synergistic effect compared to the combined level of growth suppression achieved by single compound of HDAC inhibitor, vorinostat, and PI3K inhibitor, GDC-0941 [133]. CUDC907 has proved to be therapeutic against B cell lymphoma by decreasing MYC protein levels [134]. The effect of dual inhibition synergistically induced apoptosis of MYC-altered cells in diffuse large B-cell lymphoma (DLBCL) [135]. For cancer cells that developed resistance to PI3K inhibition through alternative pathway activation, concurrent inhibition of HDAC can down-regulate other signaling proteins and circumvent treatment resistance. This potential benefit of dual inhibition was supported by an in vitro finding which demonstrated that administration of HDAC inhibitor successfully overcame resistance to mTOR inhibitor in lymphoma cells [136]. An in vivo study has also revealed that dual inhibition of PI3K and HDAC can defeat cancer resistance to platinum-based treatment by suppressing multidrug resistance transporters and DNA repairs [137]. The first phase I trial of CUDC-907 for the treatment of relapsed/refractory lymphoma achieved two CRs and three PRs in patients with DLBCL [138]. There is an actively ongoing phase I trial of CUDC-907 for the patients with advanced or relapsed solid tumors (NCT02307240), and another phase I trial for the patients with metastatic or locally advanced thyroid cancer (NCT03002623).

## Inhibition of PI3K pathway and immune system

It has been well known that inhibitors of mTOR, such as sirolimus, modulate immune system. Clinically, they have been used as immune suppressive agents to prevent rejection for patients who had undergone organ transplant. In fact, PI3K family controls many aspects of cell development, differentiation and function in both innate and adaptive immune system [139]. Especially, PI3Kγ and PI3Kδ are highly expressed in all subtypes of leukocyte, and inhibition of PI3Kγ suppressed progression of breast cancer in an animal model by inhibiting tumor inflammation and myeloid cell-mediated angiogenesis [140]. Furthermore, it has been revealed that PI3Kγ in macrophage has a critical role in the interplay between immune stimulation and suppression during inflammation or cancer development [141]. Class I PI3K signaling becomes activated by antigen receptors expressed by T and B cells, altering adaptive immune system. Therefore, inhibition of PI3Kδ dampens regulatory T cells, enhances activity of cytotoxic T cells and induces tumor regression as shown in animal models of melanoma, lung cancer, thymoma and breast cancer [142]. Various mutations in genes encoding PI3Kδ may as well lead to immunodeficiency syndromes [143].

Immune checkpoint inhibitors such as anti-programmed death 1 (anti-PD1) antibodies have demonstrated remarkable activities in HNSCC [144, 145]. Interestingly, the level of immune checkpoint ligands such as programmed death ligand 1 (PD-L1) appears to be regulated by the PI3K-Akt-mTOR pathway: inhibition of PI3K, Akt or mTOR decreased expression of PD-L1 in a non-small cell lung cancer model in vitro and in vivo [146]. Furthermore, combination of PI3Kγ blockade and immune checkpoint blockade with anti-PD1 therapy induced a synergistic growth inhibitory effect in animal models of both HPV-positive and negative HNSCC [141]. In this study, the authors showed that PI3Kγ in macrophages plays a key role in inducing immune suppression by inhibiting NFκB pathway. Inhibition of PI3Kγ in macrophages, therefore, stimulated NFκB activation and promoted an immunostimulatory transcriptional program, restoring T cell activation. Another report suggests that PI3K-Akt pathway activation may mediate Tim-3 expression in HNSCC, which is associated with more exhausted phenotype of tumor infiltrating lymphocytes, and cause resistance to immune checkpoint blockade [147]. However, the role of PI3K pathway in cancer immunology needs to be clinically investigated further. There are phase I trials of combining PI3Kδ inhibitor (INCB050465) with pembrolizumab in advanced solid tumors (NCT02646748), and combining PI3Kβ inhibitor (GSK2636771) with pembrolizumab in advanced melanoma (NCT03131908). With recent approvals of immune checkpoint inhibitors for the treatment of R/M HNSCC, effects of adding PI3K inhibitors to immune checkpoint inhibitors will be further explored.

## Conclusions

PI3K plays a key role in the progression of HNSCC and development of resistance against cetuximab. Genomic alterations affecting PI3K are common among both HPV-positive and HPV-negative diseases and serve as an attractive target for the treatment of HNSCC. Early clinical trials evaluating PI3K inhibitors have shown disappointing results, but further evaluation with more potent agents and careful patient selection might lead to development of effective PI3K inhibitors in HNSCC. In light of recent success of immune checkpoint inhibitors, potential impacts of PI3K inhibition on immune system should be considered in the future development of PI3K-targeted therapy.

## Abbreviations

anti-PD1: anti-programmed death 1
BH: Breakpoint cluster region-homology
CR: Complete response
DLBCL: Diffuse large B-cell lymphoma
EGFR: Epidermal growth factor receptor
Erk: Extracellular signal-regulated kinase
FKBP12: 12 kDa FK506-binding protein
GAP: GTPase activating protein
GEF: Guanine nucleotide exchange factor
GPCR: G protein-coupled receptor
HDAC: Histone deacetylase
HNSCC: Head and neck squamous cell carcinoma
HPV: Human papillomavirus
IGF-1R: Insulin-like growth factor 1 receptor
IHC: Immunohistochemistry
IRS-1: Insulin receptor substrate 1
MEK: MAPK(mitogen-activated protein kinase)/Erk kinase
mTOR: mechanistic target of rapamycin
mTORC1: mTOR complex1
NET: Neuroendocrine tumor
OS: Overall survival
PDGFR: Platelet-derived growth factor receptor
PDK1: Phosphoinositide-dependent kinase 1
PD-L1: Programmed death-ligand 1
PFS: Progression-free survival
PH: Pleckstrin homology
PI: Phosphatidylinositol
PI-3,4-P2: Phosphatidylinositol 3,4-bisphosphate
PI3K: Phosphoinositide 3-kinase
PI-3-P: Phosphatidylinositol 3-phosphate
PIP: Phosphatidylinositol 4-phosphate
PIP2: Phosphatidylinositol 4,5-bisphosphate
PIP3: Phosphatidylinositol 3,4,5-triphosphate
PKB: Protein kinase B
PR: Partial response
PTEN: Phosphatase and tensin homolog
Ral: Ras-like protein
Rheb: Ras homolog enriched in brain
SD: Stable disease
SH: Src homology
TCGA: The cancer genome atlas
TSC: Tuberous sclerosis complex
